# Particulate Matter 2.5 Exposure and Self-Reported Use of Wood Stoves and Other Indoor Combustion Sources in Urban Nonsmoking Homes in Norway

**DOI:** 10.1371/journal.pone.0166440

**Published:** 2016-11-17

**Authors:** Annah B. Wyss, Anna Ciesielski Jones, Anette K. Bølling, Grace E. Kissling, Ryan Chartier, Hans Jørgen Dahlman, Charles E. Rodes, Janet Archer, Jonathan Thornburg, Per E. Schwarze, Stephanie J. London

**Affiliations:** 1 Division of Intramural Research, National Institute of Environmental Health Sciences, National Institutes of Health, U.S. Department of Health and Human Services, Research Triangle Park, North Carolina, United States of America; 2 Social & Scientific Systems, Inc., Durham, North Carolina, United States of America; 3 Department of Air Pollution and Noise, Domain for Infection Control and Environmental Health, Norwegian Institute of Public Health, Oslo, Norway; 4 Research Triangle Institute International, Research Triangle Park, North Carolina, United States of America; Utah State University, UNITED STATES

## Abstract

Few studies have examined particulate matter (PM) exposure from self-reported use of wood stoves and other indoor combustion sources in urban settings in developed countries. We measured concentrations of indoor PM < 2.5 microns (PM_2.5_) for one week with the MicroPEM^™^ nephelometer in 36 households in the greater Oslo, Norway metropolitan area. We examined indoor PM_2.5_ levels in relation to use of wood stoves and other combustion sources during a 7 day monitoring period using mixed effects linear models with adjustment for ambient PM_2.5_ levels. Mean hourly indoor PM_2.5_ concentrations were higher (p = 0.04) for the 14 homes with wood stove use (15.6 μg/m^3^) than for the 22 homes without (12.6 μg/m^3^). Moreover, mean hourly PM_2.5_ was higher (p = 0.001) for use of wood stoves made before 1997 (6 homes, 20.2 μg/m^3^), when wood stove emission limits were instituted in Norway, compared to newer wood stoves (8 homes, 11.9 μg/m^3^) which had mean hourly values similar to control homes. Increased PM_2.5_ levels during diary-reported burning of candles was detected independently of concomitant wood stove use. These results suggest that self-reported use of wood stoves, particularly older stoves, and other combustion sources, such as candles, are associated with indoor PM_2.5_ measurements in an urban population from a high income country.

## Introduction

Wood is a common heating fuel in some developed countries such as Norway where 12% of the population uses wood as a main source of heating [1]. Previous studies in developed countries, conducted mostly in rural areas, have shown wood stove use to be an important source of indoor PM_2.5_ (particulate matter with aerodynamic diameter < 2.5 μm) [2–7]. Further, studies of wood stove exchange programs in rural regions of the United States have suggested that improvements in stove design may lead to reductions in both indoor and outdoor PM_2.5_ levels [8–12]. There are few data in urban areas in developed countries. Questionnaires are a convenient way to assess exposures and their effects on health outcomes in large population-based studies. However, there are few data on how self-reported use of wood stoves predicts particulate matter (PM) exposure in the home. The relationship between indoor PM_2.5_ and self-reports of other indoor sources of fine particulates, such as the burning of candles or cooking, is also not well described.

To assess whether the self-reported use of wood stoves and other household activities are associated with indoor exposure to fine particles in an urban setting in a developed country, we performed a pilot study in nonsmoking households in the greater metropolitan area of Oslo, Norway. Participants recorded activities that could generate PM in an hourly diary over a seven day period, including whether wood or candles were burned or food was fried. The MicroPEM^™^ device was used to collect real-time PM_2.5_ data during the same time period. Data were analyzed to determine whether self-reported activities in the homes were associated with indoor PM_2.5_ levels.

## Materials and Methods

### Study Design

Participants were employees at the Norwegian Institute of Public Health in Oslo, Norway who either reported having a wood stove as the main source of heating in their home (N = 18) or reported having no wood stove (N = 23). Measurements of fine particulates within participants’ homes were made between November 27, 2012 and February 17, 2013 using the MicroPEM^™^ [2,13].

The MicroPEM^™^ uses a light-scattering nephelometer fitted with a size selective dual impactor stage to measure the real time concentration of particles with an aerodynamic diameter of less than 2.5 microns. The device also uses an internal Teflon filter to collect particles during the sampling for gravimetric analysis [14]. The filters were pre-weighed at RTI International (Research Triangle Park, NC) and then packed in airtight cassettes for transport to the study site in Norway. Before participants were given the MicroPEM^™^ monitor to take home, study staff inserted the filters and used a calibration device (Mass Flowmeter 4140, TSI, Shoreview, MN) to set the air flow rate to 0.50 liters/min.

Participants were instructed to attach the monitors to a 1.12 meter high stand located in the main living space of their homes. This height corresponded to estimated seated breathing height, which was based on the average population height of 1.73 meters, according to Norway’s Directorate of Health [15]. Once participants turned the monitor on, measurements were taken for 7-days according to the following cycle: 20 second sampling period followed by 100 seconds without sampling. A sampling schedule of 20/100 enabled 7 days of data collection on a single set of AA batteries. Unpublished RTI data showed that this power cycling schedule detected brief PM peak concentrations 99% of the time.

Participants also filled out a questionnaire about their homes and stoves, including the production year, brand, and model of the stove. If the exact year of the stove was not known, participants recorded whether it was manufactured before or after 1997, when emission limits for new stoves were instituted in Norway and a stove exchange program was conducted in Oslo. The questionnaire also queried the type of wood fuel—log, pellet, other—used in the stove.

In addition, participants kept a daily diary documenting for each hour of the day whether the following activities were performed: wood burned in the wood stove, wood burned in the fireplace, candles burned, food fried, smoking by anyone including you, smoke for other reasons (e.g., burning food or accidental fire), or windows open. The daily diary was completed over the same dates as the 7-day monitoring period.

At the end of the monitoring period, staff removed the filters from the monitors and stored them in a refrigerator at 4°C until they were returned to RTI and weighed. The weight and filter measurements, along with the weights of the blank filters, were used to calibrate the individual nephelometer and generate estimates of the real-time PM_2.5_ concentration in μg/m^3^.

In two homes measurements were not completed due to monitor malfunction. Another three homes were excluded due to unreliable filter weights; two homes with wood stoves had filter post-weights less than pre-weights and one home without a wood stove had a filter net-weight of 0.9 μg, which was less than the minimum detection limit of 1 μg for the gravimetric analysis method. Thus, data were complete for 36 homes and were used for the main analysis.

In three of the homes the monitor was not turned off at the end of seven days. Because the questionnaires provided space to record activities for only seven days, activities for the hours of sampling done beyond the end of the seventh day were not recorded for these three homes. While this extra PM monitoring time was included in the filter weights and has corresponding nephelometer data, the lack of associated activity data meant that these hours (80 hours) were not included in the analysis. In another seven homes (four with wood stoves and 3 without), the monitor shut down before the end of seven days due to low batteries. The median length (range) of the monitoring period was 7.0 days (range, 5.6–9.9 days) for homes using wood stoves and 7.0 days (range, 5.0–7.7 days) for homes not using wood stoves.

Of the sixteen homes that self-identified as the ‘wood stove’ group and had usable data, two of the homes did not use their wood stoves during the sampling period. Thus, they were included in the ‘non-wood stove use in the past 7 days’ group.

To provide an estimate of ambient PM_2.5_ levels, we obtained hourly levels (in μg/m^3^) from an urban background monitor in Oslo (Sofienbergparken) for the period November 1, 2012 to February 28, 2013. This station has annual average levels of 11 μg/m^3^. The outdoor temperature during the monitoring days ranged from -17.2°C to 5.4°C (median daily average: -6°C, interquartile range: -8.9 to -2.1°C). These temperatures were recorded at the Hovin, Alna, and Blindern meteorological stations in Oslo.

The NIH Office of Human Subjects Research determined that this study was exempt from IRB review.

### Statistical Analysis

Mean hourly PM_2.5_ concentrations across the seven day monitoring period were analyzed by repeated measures analyses of covariance, comparing study group (wood stove use in the past 7 days versus not) and reported time-periods for the following potential PM-generating activities (yes versus no): burning of wood in stove, burning of wood in fireplace, burning of candles, frying of food, smoking in the home, and smoke in home for other reasons. Among households with wood stoves, mean hourly PM_2.5_ concentrations were also analyzed by the year the wood stove was manufactured (1996 and before versus 1997 and after). Ambient particulate concentrations were included as a covariate in all statistical models. Repeated measures analysis of covariance was also used to regress log-transformed PM_2.5_ levels on all reported conditions concomitantly (i.e. the model included wood stove use, fireplace use, candle burning, frying food, other smoke, and opening windows). In addition, this full model included interactions terms between wood stove use and each of the other conditions to assess whether each respective effect was dependent on wood stove use. Household was included in the full statistical model as a random effect to account for the clustering of readings within each of the houses. To estimate the change in mean hourly PM_2.5_ when the activity was present, we calculated the model-estimated mean log_10_(PM_2.5_ + 0.01) when the activity was present and when it was absent, holding all other conditions constant, and exponentiated these to obtain estimated levels of PM_2.5_ for when each activity was present and when it was absent. We then took the difference between estimated PM_2.5_ when the activity was present and when it was absent to obtain the change. In the case of interaction terms, we took the difference between when both activities were present and when both activities were absent. We used PROC MIXED in SAS (Version 9.3 Cary, NC, USA) to conduct these analyses (see S1 File for code). Statistical significance was defined as p<0.05.

## Results

Over the entire 7-day monitoring period, the 14 households that used wood stoves burned wood for a median of 21.5 hours (range = 8.5–44.8, Table 1). Among households that used wood stoves, all reported frying food at least once during the study period with a median of 4.5 hours spent frying food during the week. In addition, 79% of households that used wood stoves reported burning candles for a median of 5 hours during the week. Among the 22 households without wood stove use, 77% reported frying food (median hours spent frying food during study period was 5) and 73% reported using candles (median hours spent burning candles during study period was 8.5). Half of the households with wood stove use in the past 7 days (50%) and about three-fourths (73%) of those without wood stove use reported opening windows during the study period. Fireplace use and smoke for other reasons (e.g., burning food or other accidental fires) was not commonly reported. None of the participants reported any smoking of cigarettes or cigars inside the home during the sampling period.

**Table 1:** Percent of Participating Norwegian Homes (N = 36) With Reporting of Activities that could Generate Particulate Matter and Median Number of Hours Activities were Performed During a 7 Day Period.

	Stove Users (n = 14)	Stove Non-Users (n = 22)
Number of homes with stoves older than 1997	6	-
Number of hours burned wood in stove ^0^	21.5 (8.5–44.8)	-
% of homes with wood burned in fireplace	21%	18%
Number of hours burned among fireplace users ^0^	3.0 (2.5–3.5)	12.0 (4.3–20.3)
% of homes with candle burning	79%	73%
Number of hours burned among users ^0^	5.0 (3.0–8.0)	8.5 (4.0–16.3)
% of homes with frying of food	100%	77%
Number of hours fried among users ^0^	4.5 (3.0–6.8)	5.0 (4.0–7.0)
% of homes with any smoking in the home	0%	0%
% of homes reporting another smoke source^1^	14%	14%
Number of hours with other smoke among users ^0^	5.0 (3.0–7.0)	1.0 (1.0–1.0)
% of homes reporting hours with windows open	50%	73%
Number of hours with windows open ^0^	7.0 (3.5–30.5)	6.0 (1.8–22.8)

PM_2.5_ = particulate matter with aerodynamic diameter < 2.5 μm

^0^Median (Interquartile Range)

^1^Other smoke sources included food burning and other accidental fires

**Fig 1:**
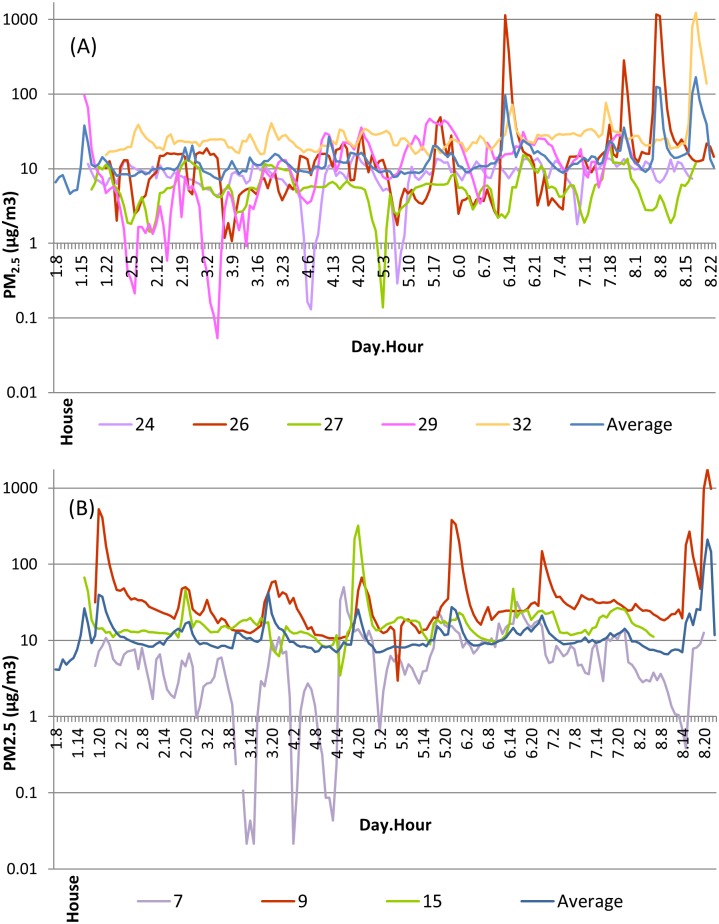
Mean Hourly PM_2.5_ Concentrations Across Study Time Period for Select Homes With Wood Stove Use (Panel A) and Without Wood Stove Use (Panel B). Mean hourly PM_2.5_ concentrations for homes selected for exhibiting peaks in PM_2.5_ concentrations (2 homes with wood stove use and 2 homes without wood stove use) or dips in PM_2.5_ concentrations (3 homes with wood stove use and 1 home without a wood stove use). Hourly PM_2.5_ concentrations averaged across homes with wood stove use (N = 14 homes) and without wood stove use (N = 22 homes) are also displayed. S1 Fig shows mean hourly PM_2.5_ concentrations for every home.

S1 Fig shows mean hourly PM_2.5_ concentrations for each individual home with and without wood stove use. Among homes with wood stove use, the highest peak occurred at day 8 hours 17 to 19 and corresponds to a home in which frying of food during that time was reported (Fig 1, Panel A). Two other noticeable peaks at day 6 hour 13 and day 8 hours 7 to 8 correspond to a different home which reported burning wood in a wood stove and smoke for other reasons during those hours (Fig 1, Panel A). Three noticeable dips occurred at day 3 hour 5, day 4 hours 6 to 7, and day 5 hour 3 and correspond to three different homes (Fig 1, Panel A). These near zero PM_2.5_ concentrations occurred during periods when those homes reported no PM-generating activities, though one home reported opening windows. Among homes without wood stove use, high peaks occurred at day 1 hours 20 to 21 and day 8 hours 20 to 22 and correspond to the same home which reported frying of food and opening of windows the hour prior (Fig 1, Panel B). Another noticeable peak at day 3 hour 19 corresponds to a home which fried food, used a fireplace, used candles, had smoke for other reasons, and opened windows around that time (Fig 1, Panel B). Three noticeable dips occurred at day 3 hours 11 to 15, day 4 hours 2 and hours 11 to13 and correspond to the same home which reported opening windows but no other activities (Fig 1, Panel B).

Adjusting for ambient concentrations, mean hourly PM_2.5_ concentrations were higher (p = 0.04) in homes with wood stove use in the past 7 days [15.6 μg/m^3^ (SD = 54.1, range = 0–1,225)] than in the homes without [12.6 μg/m^3^ (SD = 42.8, range = 0–1,765)]. We also found that PM_2.5_ was higher (p = 0.001) from wood burning in stoves that were made before 1997 (N = 6, mean = 20.2 μg/m^3^, SD = 80.2, range = 0–1,225) compared with newer stoves (N = 8, mean = 11.9 μg/m^3^, SD = 11.7, range = 0–222).

When we analyzed data according to hours when specific combustion sources were reported (yes versus no) (Table 2), we found that mean hourly PM_2.5_ concentrations were higher during use of a wood stove (p = 0.02), use of candles (p = 0.02), and frying food (p = 0.02). In addition, mean hourly PM_2.5_ concentrations were higher during use of wood stove or fireplace (p = 0.009); however, this result was likely driven by wood stove use and not by fireplace use since the mean hourly PM_2.5_ concentrations did not vary statistically by the uncommon use of fireplaces alone (p = 0.3).

**Table 2:** Mean Hourly PM_2.5_ Levels (μg/m^3^) Recorded by Monitor During Hours With and Without Activities that Could Generate Particulate Matter.

Activity	With Activity		Without Activity		p-value^0^
	Total Number of Hours	Mean PM_2.5_ (SE)	Total Number of Hours	Mean PM_2.5_ (SE)	
Wood stove in use	399	26.4 (5.0)	5,574	12.9 (0.5)	0.02
Wood stove or fireplace in use	450	26.3 (4.6)	5,523	12.7 (0.5)	0.009
Candles burning	293	20.3 (2.1)	5,680	13.4 (0.6)	0.02
Fireplace in use	57	25.4 (9.5)	5,916	13.6 (0.6)	0.3
Frying food	172	34.5 (8.6)	5,801	13.1 (0.6)	0.02
Other activity producing smoke^1^	12	268.4 (126.0)	5,961	13.2 (0.5)	0.07

PM_2.5_ = particulate matter with aerodynamic diameter < 2.5 μm; SE = standard error

^0^P-values from repeated measures analysis of covariance regressing PM_2.5_ levels on each activity separately, adjusted for ambient PM_2.5_ concentrations.

^1^Other smoke sources included food burning and other accidental fires

When all conditions were modeled concomitantly, wood stove use, fireplace use, candle burning, frying of food, other activities producing smoke, and ambient particulate concentration each contributed significantly to the variation in PM_2.5_ concentrations based on the Type III Sums of Squares (p≤0.002, Table 3). In contrast, reported opening of windows did not contribute significantly to the variation in PM_2.5_ concentrations (p = 0.6). Although, we observed a negative significant interaction term between wood stove use and frying of food and a positive significant interaction term between wood stove use and other sources of smoke, these interactions may simply reflect higher mean PM levels during the relatively little time spent frying food when the wood stove was off or with other smoke sources when the wood stove was on, respectively. Parameter estimates from the model presented in Table 3 represent log-transformed mean hourly PM_2.5_ levels, and should be interpreted based on the presence or absence of other conditions. Table 3 also shows the estimated change in mean hourly PM_2.5_ levels in μg/m^3^ in the presence of each activity, holding all other sources constant. As an example, consider the combinations of wood stove use and candle burning. Holding all other conditions constant, mean hourly PM_2.5_ levels were 77 μg/m^3^ higher when wood stoves were used. Similarly, PM_2.5_ levels were 15 μg/m^3^ higher when candles were burned. When both wood stoves and candles were used (again holding all other sources constant), PM_2.5_ levels were 91 μg/m^3^ higher compared to when neither were used.

**Table 3:** Linear Model of Log-transformed Mean Hourly PM_2.5_ Levels Including Reported Particulate Matter Generating Activities Concomitantly.

	Type III Sums of Squares	Parameter Estimates from Linear Model of Log-transformed PM_2.5_ Levels^0^	Change in Mean Hourly PM_2.5_ Due to Activity^1^
Activity	P-value	β(SE)	μg/m^3^
Wood stove use	0.001	0.209 (0.053)	77
Fireplace use	0.0007	0.314 (0.043)	28
Candles burning	0.0002	0.159 (0.020)	15
Frying food	0.002	0.223 (0.025)	12
Other activity producing smoke^2^	<0.0001	0.386 (0.099)	171
Windows open	0.6	0.019 (0.019)	-4
Ambient particulate matter	<0.0001	0.006 (0.0003)	1
Household^3^	-	-	-
Wood stove use*Fireplace use	0.2	-0.183 (0.131)	102
Wood stove use*Candle burning	0.2	-0.087 (0.062)	91
Wood stove use*Frying food	<0.0001	-0.249 (0.063)	80
Wood stove use*Other activities producing smoke	<0.0001	1.360 (0.197)	768
Wood stove use*Windows open	0.4	-0.096 (0.117)	68
Wood stove use*Ambient particulate matter	0.4	-0.001 (0.001)	1
Wood stove use*Household^3^	-	-	-

PM_2.5_ = particulate matter with aerodynamic diameter < 2.5 μm; SE = standard error

^0^Estimate for yes vs. no for each activity adjusting for all other activities in table

^1^Change in mean hourly PM_2.5_ for yes vs. no for each activity, holding all other activities in table constant; change in mean hourly PM_2.5_ for each 1 μg/m^3^ increase in ambient particulate matter; for interaction terms, change in mean hourly PM_2.5_ for both activities vs. neither activity, holding all other activities in the table constant. S1 Table contains the least squares means estimates used to calculate the change in mean hourly PM_2.5_ due to each activity.

^2^Other smoke sources included food burning and other accidental fires

^3^Random effects variance for household is 0.02 and for wood stove use*household is 0.01.

## Discussion

In this study of 36 homes in the Oslo, Norway metropolitan area, indoor levels of PM_2.5_ were significantly higher in homes where wood stove use was reported than in homes without reported use. Mean hourly PM_2.5_ concentrations were 15.6 μg/m^3^ among homes with reported use during the monitoring period compared to 12.6 μg/m^3^ in homes without. This difference was seen even though the number of hours that wood was burned was relatively limited, and persisted after adjustment for outdoor ambient levels.

Previous studies on wood stoves and particulate matter in developed countries are few and have primarily focused on rural rather than urban areas. Among European studies, 24-hour mean PM_2.5_ concentrations were 7.7 μg/m^3^ in 22 homes that burned wood in Scotland and Ireland [7] and 22.1 μg/m^3^ in 7 homes that burned wood in Germany [5]. Additionally, a study in Sweden reported median indoor PM_2.5_ concentrations of 12.0 μg/m^3^ for 13 homes that burned wood and 9.5 μg/m^3^ for 10 homes that did not burn wood [4]. This was the only previous study that included control homes without wood stoves, and found that PM_2.5_ concentrations were not statistically different between homes with and without wood stoves [4]. In a study from the Northwest region of the US, including Alaska, the mean daily PM_2.5_ concentration was 29 μg/m^3^ among 96 homes which used wood stoves [6] and a series of studies in the Rocky Mountain region reported mean daily PM_2.5_ concentrations around 20 to 40 μg/m^3^ in homes, depending on the town and types of stoves [8–12]. In addition, a study of 32 individuals in Delaware reported a mean residential concentration of 11.7 μg/m^3^ [2]. For reference, The World Health Organization (WHO) recommendation for 24-hour average indoor PM_2.5_ levels is the same as the outdoor guideline of 25 μg/m^3^ [16,17].

Despite our modest sample size, we found that PM_2.5_ levels were significantly higher for stoves manufactured prior to 1997 (20.2 μg/m^3^) compared to stoves after 1997 (11.9 μg/m^3^) when standards changed in Norway to reduce exposures. Moreover, the hourly mean values in homes with newer stoves (11.9 μg/m^3^) were approximately equal to the hourly mean values in control homes (12.6 μg/m^3^), suggesting that the use of older stoves is an important source of indoor PM_2.5_ in houses in which they are used. In the United States, intervention studies evaluating the impact of replacing older wood stoves with newer models meeting Environmental Protection Agency (EPA) standards have noted reductions in PM_2.5_ concentrations of varying amounts [8–12]. For example, an initial study of a stove change-out program in Libby, Montana reported mean PM_2.5_ concentrations in participating homes decreased by over 70% [12]. However, a follow-up study found an attenuated mean reduction closer to 50%, with some homes showing no reduction, suggesting, as we also found, that sources other than wood stoves contribute to indoor PM_2.5_ [8].

We detected PM_2.5_ exposure related to reported burning of candles, independent of concomitant burning of wood in the home. Candle burning is popular during the winter in Norway and other Scandinavian countries and was very common in our study households (>73%). A study of homes with wood stoves in Montana also found that use of candles was associated with higher indoor PM_2.5_ concentrations [6]. We also identified PM_2.5_ peaks from frying of food. All of the residents of the households in our study were nonsmokers and no smoking was reported in the homes. Because previous studies have found high PM_2.5_ concentrations in homes with resident smokers [7], the absence of smokers may have aided our ability to detect effects of wood burning and other activities, such as burning candles.

Our study builds on previous research by investigating associations between wood stoves and particulate matter in an urban setting of a developed country, by including a comparison group of homes without wood stoves, by collecting frequent measures of PM_2.5_ concentrations over a week using a nephelometer, and by ascertaining the occurrence of multiple activities involving combustion sources through diaries. Inclusion of both self-reported activities and real-time PM_2.5_ concentrations in the study allowed us to evaluate whether various activities influenced measured PM_2.5_ levels.

In Norway, it is common for both parents of a family to work during the day. Therefore, wood stove use, candle burning, and food frying typically occurs for only a few hours in the evening. Norwegian homes are well insulated and primarily rely on electrical heating systems (e.g., electrical wall units, heat pumps or water based radiators linked to heat pumps or district heating facilities) that do not generate appreciable indoor PM levels.

Our data were adjusted for hourly outdoor ambient particulate matter concentrations. While it would have been preferable to adjust for ambient concentrations collected at each home site, we were not able to collect these data and thus relied on data from a single urban monitor. Using measurements from this monitor, located closer to the city center, may have slightly overestimated ambient PM_2.5_ levels at homes near the city limits. However, studies conducted in Detroit found that PM_2.5_ measurements recorded by a central monitor were correlated with measurements taken from several monitors located in other areas of the city, suggesting that a single well-placed monitor may capture representative PM_2.5_ levels for a large metropolitan area [18,19]. Further, ambient particulate matter would not explain away the findings we observed. If PM_2.5_ levels peaked in non-wood stove homes during the time neighboring homes used wood stoves, such an event would lead to an underestimation, not an overestimation, of the effect of indoor wood burning on the difference in PM_2.5_ levels between homes with and without wood stove use.

While PM_2.5_ levels directly following the conclusion of combustion activities may remain elevated due to smoldering or delayed clearance of smoke, we could not fully consider such lag effects. PM levels were not collected continuously (20 second sampling periods followed by 100 seconds without sampling) and participants reported activities in coarser 1-hour increments. Also, the autocorrelation between hourly average PM levels and those in the prior hour was high. Therefore, inclusion of lag effects would likely result in over-adjustment of our models.

Because our study population consisted of well-educated volunteers of similar socioeconomic status from the Norwegian Public Health Institute, results might not be generalizable to studies based in very different populations. However, our study demonstrates that self-reported wood stove use and other activities such as burning of candles contribute significantly to hourly indoor PM_2.5_ levels. While nephelometers are needed for detailed studies of time resolved activities and PM_2.5_ levels, our findings may facilitate the development and refinement of future questionnaires to assess the health effects of general household exposures shown here to be associated with indoor PM_2.5_ levels. In addition, these results may also inform future studies aimed at developing predictive models of PM exposure from multiple indoor sources, including wood stoves.

## Supporting Information

S1 Fig Mean hourly PM_2.5_ concentrations for every home. (TIF)

S1 File SAS code for PROC MIXED. (DOC)

S2 File Mean Hourly PM_2.5_ Concentrations and Hourly Activities. (XLSX)

S1 Table The least squares means estimates used to calculate the change in mean hourly PM_2.5_ due to each activity. Least Squares Means Estimates from Linear Model of Log-transformed Mean Hourly PM_2.5_ Levels Including Reported Particulate Matter Generating Activities Concomitantly.
(DOC)
