# Supplementary figures and images for: Particulate Matter 2.5 Exposure and Self-Reported Use of Wood Stoves and Other Indoor Combustion Sources in Urban Nonsmoking Homes in Norway
Source: PLoS One. 2016 Nov 17;11(11):e0166440. doi: 10.1371/journal.pone.0166440 (PMC5113953; doi:10.1371/journal.pone.0166440)

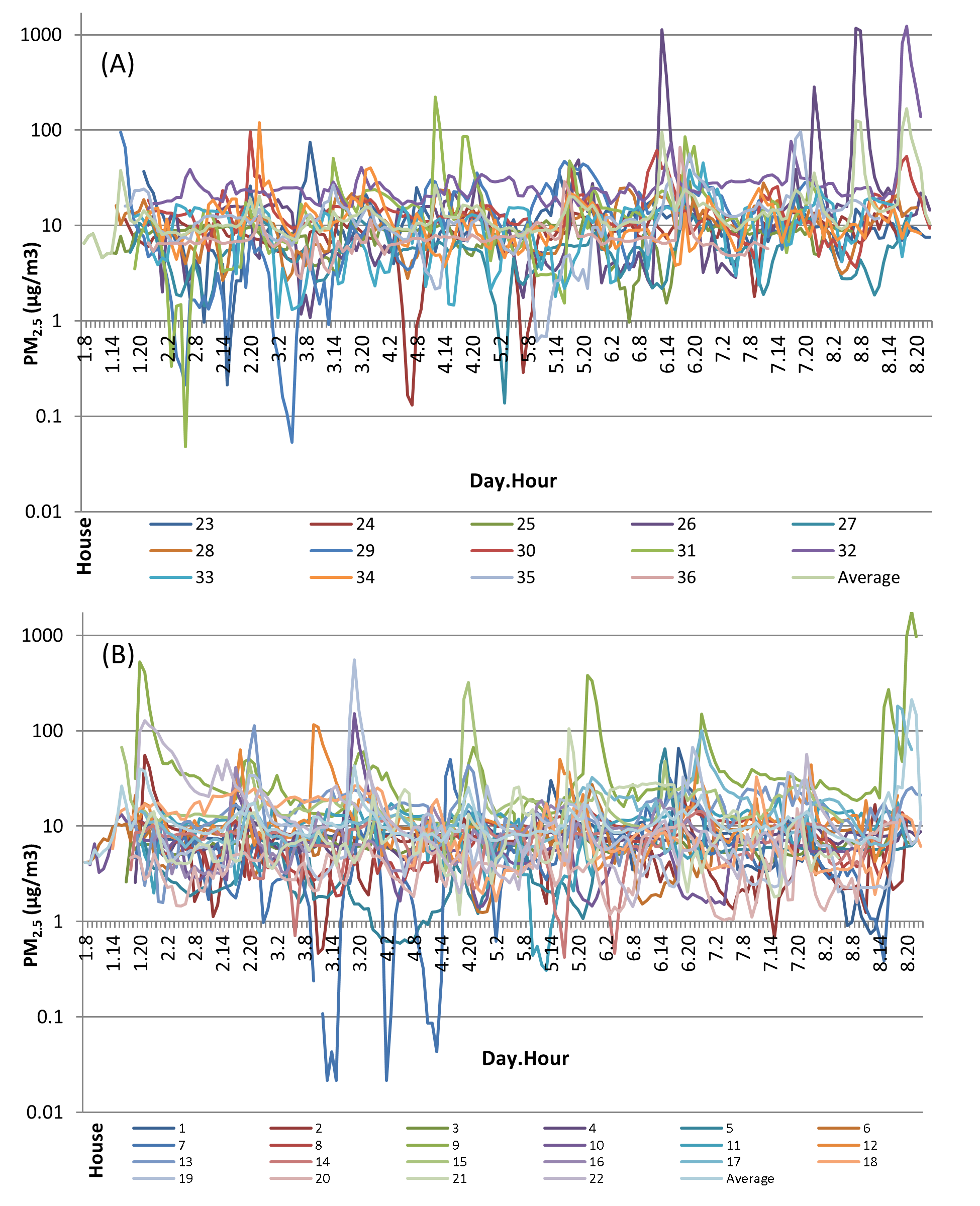

Supplement: S1 Fig — (TIF) [file pone.0166440.s001.tif]
