# Supplementary material for: Particulate Matter 2.5 Exposure and Self-Reported Use of Wood Stoves and Other Indoor Combustion Sources in Urban Nonsmoking Homes in Norway
Source: PLoS One. 2016 Nov 17;11(11):e0166440. doi: 10.1371/journal.pone.0166440 (PMC5113953; doi:10.1371/journal.pone.0166440)
Supplement: S1 File — (DOC) [file pone.0166440.s002.doc]

**S1 File. SAS Code**

* Mean PM2.5 concentration by wood stove use;

**proc** **mixed** data=work.hourlyPMlog1;

class Stove;

model PMhourly = Ambient Stove /ddfm=satterth;

repeated / group=Stove;

title 'proc mixed for Stove, Adjusted for Ambient';

**run**;

* Mean PM2.5 concentration by wood stove year;

**proc** **mixed** data=work.hourlyPMlog1;

class StoveYr;

model PMhourly = Ambient StoveYr /ddfm=satterth;

repeated / group=StoveYr;

title 'proc mixed for Stove Year, Adjusted for Ambient';

where StoveYr = '1996before' or StoveYr = '1997after';

**run**;

* Table 2: Mean PM2.5 concentration by hours when specific conditions were met;

**proc** **mixed** data=work.hourlyPMlog1;

class WoodstoveOn;

model PMhourly = Ambient WoodstoveOn /ddfm=satterth;

repeated / group=WoodstoveOn;

**run**;

**proc** **mixed** data=work.hourlyPMlog1;

class woodORfire;

model PMhourly = Ambient woodORfire /ddfm=satterth;

repeated / group=woodORfire;

**run**;

**proc** **mixed** data=work.hourlyPMlog1;

class CandleOn;

model PMhourly = Ambient CandleOn /ddfm=satterth;

repeated / group=CandleOn;

**run**;

**proc** **mixed** data=work.hourlyPMlog1;

class FireplaceOn;

model PMhourly = Ambient FireplaceOn /ddfm=satterth;

repeated / group=FireplaceOn;

**run**;

**proc** **mixed** data=work.hourlyPMlog1;

class FriedFood;

model PMhourly = Ambient FriedFood /ddfm=satterth;

repeated / group=FriedFood;

**run**;

**proc** **mixed** data=work.hourlyPMlog1;

class OtherSmoke;

model PMhourly = Ambient OtherSmoke /ddfm=satterth;

repeated / group=OtherSmoke;

**run**;

Table 3: Linear model of log-transformed mean hourly PM2.5 levels including reported activities concomitantly;

**proc** **mixed** data=work.referent;

class WoodStoveOn FireplaceOn CandleOn FriedFood OtherSmoke WindowsOpen Household_ID;

model logPMhour=WoodStoveOn FireplaceOn CandleOn FriedFood OtherSmoke WindowsOpen ambient

WoodStoveOn*FireplaceOn WoodStoveOn*CandleOn WoodStoveOn*FriedFood WoodStoveOn*OtherSmoke WoodstoveOn*WindowsOpen

WoodstoveOn*ambient / solution ;

random Household_ID WoodstoveOn*Household_ID;

lsmeans WoodStoveOn;

lsmeans FireplaceOn;

lsmeans CandleOn;

lsmeans FriedFood;

lsmeans OtherSmoke;

lsmeans WindowsOpen;

lsmeans WoodStoveOn*FireplaceOn;

lsmeans WoodStoveOn*CandleOn;

lsmeans WoodStoveOn*FriedFood;

lsmeans WoodStoveOn*OtherSmoke;

lsmeans WoodStoveOn*WindowsOpen;

**run**;
