# Supplementary material for: Particulate Matter 2.5 Exposure and Self-Reported Use of Wood Stoves and Other Indoor Combustion Sources in Urban Nonsmoking Homes in Norway
Source: PLoS One. 2016 Nov 17;11(11):e0166440. doi: 10.1371/journal.pone.0166440 (PMC5113953; doi:10.1371/journal.pone.0166440)
Supplement: S1 Table — Least Squares Means Estimates from Linear Model of Log-transformed Mean Hourly PM2.5 Levels Including Reported Particulate Matter Generating Activities Concomitantly. (DOC) [file pone.0166440.s004.doc]

| **S1 Table. Least Squares Means Estimates from Linear Model of Log-transformed Mean Hourly PM_2.5_ Levels Including Reported Particulate Matter Generating Activities Concomitantly.** | | | | | |
| --- | --- | --- | --- | --- | --- |
|  | **Activity Reported** | **Least Squares Means Estimates** | | | **Mean Hourly PM_2.5_ Due to Activity (i.e. 10^LSMean^)** |
| **Activity** | **Yes vs. No** | **Estimate** | **SE** | **P-value** | **µg/m^3^** |
| Wood stove use | Yes | 2.022 | 0.131 | <0.0001 | 105.27 |
| Wood stove use | No | 1.456 | 0.063 | <0.0001 | 28.60 |
| Fireplace use | Yes | 1.850 | 0.093 | <0.0001 | 70.84 |
| Fireplace use | No | 1.628 | 0.068 | <0.0001 | 42.49 |
| Candles burning | Yes | 1.797 | 0.079 | <0.0001 | 62.70 |
| Candles burning | No | 1.681 | 0.074 | <0.0001 | 48.01 |
| Frying food | Yes | 1.789 | 0.079 | <0.0001 | 61.45 |
| Frying food | No | 1.690 | 0.074 | <0.0001 | 48.99 |
| Other activity producing smoke^1^ | Yes | 2.272 | 0.113 | <0.0001 | 187.20 |
| Other activity producing smoke | No | 1.206 | 0.056 | <0.0001 | 16.08 |
| Windows open | Yes | 1.725 | 0.090 | <0.0001 | 53.05 |
| Windows open | No | 1.754 | 0.070 | <0.0001 | 56.74 |
| Wood stove use*Fireplace use | Yes, Yes | 2.088 | 0.168 | <0.0001 | 122.35 |
| Wood stove use*Fireplace use | Yes, No | 1.957 | 0.117 | <0.0001 | 90.57 |
| Wood stove use*Fireplace use | No, Yes | 1.613 | 0.072 | <0.0001 | 41.02 |
| Wood stove use*Fireplace use | No, No | 1.300 | 0.060 | <0.0001 | 19.93 |
| Wood stove use*Candle burning | Yes, Yes | 2.059 | 0.139 | <0.0001 | 114.45 |
| Wood stove use*Candle burning | Yes, No | 1.986 | 0.130 | <0.0001 | 96.83 |
| Wood stove use*Candle burning | No, Yes | 1.536 | 0.064 | <0.0001 | 34.36 |
| Wood stove use*Candle burning | No, No | 1.377 | 0.062 | <0.0001 | 23.80 |
| Wood stove use*Frying food | Yes, Yes | 2.009 | 0.139 | <0.0001 | 102.16 |
| Wood stove use*Frying food | Yes, No | 2.035 | 0.130 | <0.0001 | 108.47 |
| Wood stove use*Frying food | No, Yes | 1.568 | 0.066 | <0.0001 | 36.95 |
| Wood stove use*Frying food | No, No | 1.345 | 0.062 | <0.0001 | 22.13 |
| Wood stove use*Other activities producing smoke | Yes, Yes | 2.895 | 0.197 | <0.0001 | 785.96 |
| Wood stove use*Other activities producing smoke | Yes, No | 1.149 | 0.100 | <0.0001 | 14.10 |
| Wood stove use*Other activities producing smoke | No, Yes | 1.649 | 0.105 | <0.0001 | 44.58 |
| Wood stove use*Other activities producing smoke | No, No | 1.263 | 0.041 | <0.0001 | 18.34 |
| Wood stove use*Windows open | Yes, Yes | 1.984 | 0.164 | <0.0001 | 96.29 |
| Wood stove use*Windows open | Yes, No | 2.061 | 0.120 | <0.0001 | 115.08 |
| Wood stove use*Windows open | No, Yes | 1.466 | 0.064 | <0.0001 | 29.23 |
| Wood stove use*Windows open | No, No | 1.447 | 0.062 | <0.0001 | 27.98 |
| PM_2.5_= particulate matter with aerodynamic diameter < 2.5 µm; SE=standard error | | | | | |
| ^1^Other smoke sources included food burning and other accidental fires | | | | | |
